# HMGB1-mediated autophagy promotes docetaxel resistance in human lung adenocarcinoma

**DOI:** 10.1186/1476-4598-13-165

**Published:** 2014-07-05

**Authors:** Banzhou Pan, Dongqin Chen, Jiayuan Huang, Rui Wang, Bing Feng, Haizhu Song, Longbang Chen

**Affiliations:** 1 Department of Medical Oncology, Jinling Hospital, School of Medicine, Nanjing University, Nanjing 210002, P.R. China

**Keywords:** Autophagy, High-mobility group box 1, Chemoresistance, Lung adenocarcinoma

## Abstract

**Background:**

Docetaxel resistance remains a major obstacle in the treatment of non-small cell lung cancer (NSCLC). High-mobility group box 1 (HMGB1) has been shown to promote autophagy protection in response to antitumor therapy, but the exact molecular mechanism underlying HMGB1-mediated autophagy has not been clearly defined.

**Methods:**

Lung adenocarcinoma (LAD) cells were transfected with pcDNA3.1-HMGB1 or HMGB1 shRNA, followed by docetaxel treatment. Cell viability and proliferation were tested by MTT assay and colony formation assay, respectively. Annexin V flow cytometric analysis and western blot analysis of activated caspase3 and cleaved PARP were used to evaluate apoptosis, while immunofluorescence microscopy and transmission electron microscopy were applied to assess autophagy activity. The formation of the Beclin-1-PI3K-III complex was examined by immunoprecipitation analysis. NOD/SCID mice were inoculated with docetaxel-resistant SPC-A1/DTX cells transfected with control or HMGB1 shRNA.

**Results:**

HMGB1 translocated from the nucleus to the cytoplasm in LAD cells exposed to docetaxel and acted as a positive regulator of autophagy, which inhibited apoptosis and increased drug resistance. Suppression of HMGB1 restored the sensitivity of LAD cells to docetaxel both *in vivo* and *in vitro*. Mechanistic investigation revealed that HMGB1 promoted the formation of the Beclin-1-PI3K-III complex through activating the mitogen-activated protein kinase (MEK)-extracellular signal-regulated kinase (ERK) signaling pathway, thereby regulating autophagosome formation.

**Conclusions:**

Our results demonstrated that HMGB1-regulated autophagy is a significant contributor to docetaxel resistance in LAD cells. Suppression of HMGB1 or limiting HMGB1 cytosolic translocation diminished autophagic protection in response to docetaxel in LAD cells.

## Background

Lung cancer is the leading cause of cancer-related mortality among malignancies worldwide. Lung adenocarcinoma (LAD) is the most common type of lung cancer, and accounts for approximately 30–40% of non-small cell lung cancer (NSCLC) cases [1]. In spite of significant achievement in the treatment of LAD over the last decade, the prognosis for patients with advanced disease remains poor [2]. Docetaxel, a semi-synthetic analog of paclitaxel, was granted approval as a first-line chemotherapy regimen for NSCLC [3]. However, chemoresistance remains a major obstacle constraining the clinical application of this agent.

Autophagy, a regulated process that consists of selective degradation of cellular proteins and cytoplasmic organelles, is implicated in many physiological and pathological conditions, including intracellular recycling, energy homeostasis, neurodegeneration and, importantly, cancer [4,5]. Studies have shown a paradoxical dual effect of autophagy in cancer development and progression. Inhibition of autophagy promotes tumorigenesis [4], while induction of autophagy in established tumors promotes cell survival under nutrition starvation, cellular stress and antitumor therapies [6,7]. Clarifying the mechanism that underlies and regulates these distinct functions requires further investigation [4].

High-mobility group box 1(HMGB1) is a highly conserved non-histone nuclear protein that binds DNA and promotes assembly of proteins on specific DNA targets [8]. In addition to its role in the nucleus, HMGB1 also functions in the cytoplasm as an extracellular signaling protein during inflammation, cell differentiation and tumor progression [9,10]. Upregulation of HMGB1 expression has been demonstrated in various solid tumors and hematological malignancies, such as breast cancer and lymphoma [11,12]. Cytosolic translocation and HMGB1 release from tumor cells in response to chemotherapy and radiotherapy is a vital characteristic of the disordered tumor microenvironment [13,14]. Modification of HMGB1 localization has been shown to link autophagy and apoptosis [8], but the exact molecular mechanism of HMGB1-mediated autophagy in tumor therapy has not been clearly defined.

In this study, we found that cytosolic HMGB1 expression and associated autophagy levels increased rapidly in response to docetaxel treatment in LAD cells. Downregulation of cytosolic HMGB1 levels limited docetaxel-induced autophagic protection, which enhanced chemosensitivity of parental LAD cells and even resensitized docetaxel-resistant LAD cells. Furthermore, our data verified a role for HMGB1 in the regulation of the Beclin1-PI3K-III complex through the mitogen-activated protein kinase (MEK)-extracellular signal-regulated kinase (ERK) pathway. Together our data supports the notion that HMGB1 represents a suitable target when combined with conventional chemotherapies in LAD.

## Results

### Docetaxel induced a cytoprotective autophagy that protects LAD cells from apoptosis

**Figure 1:**
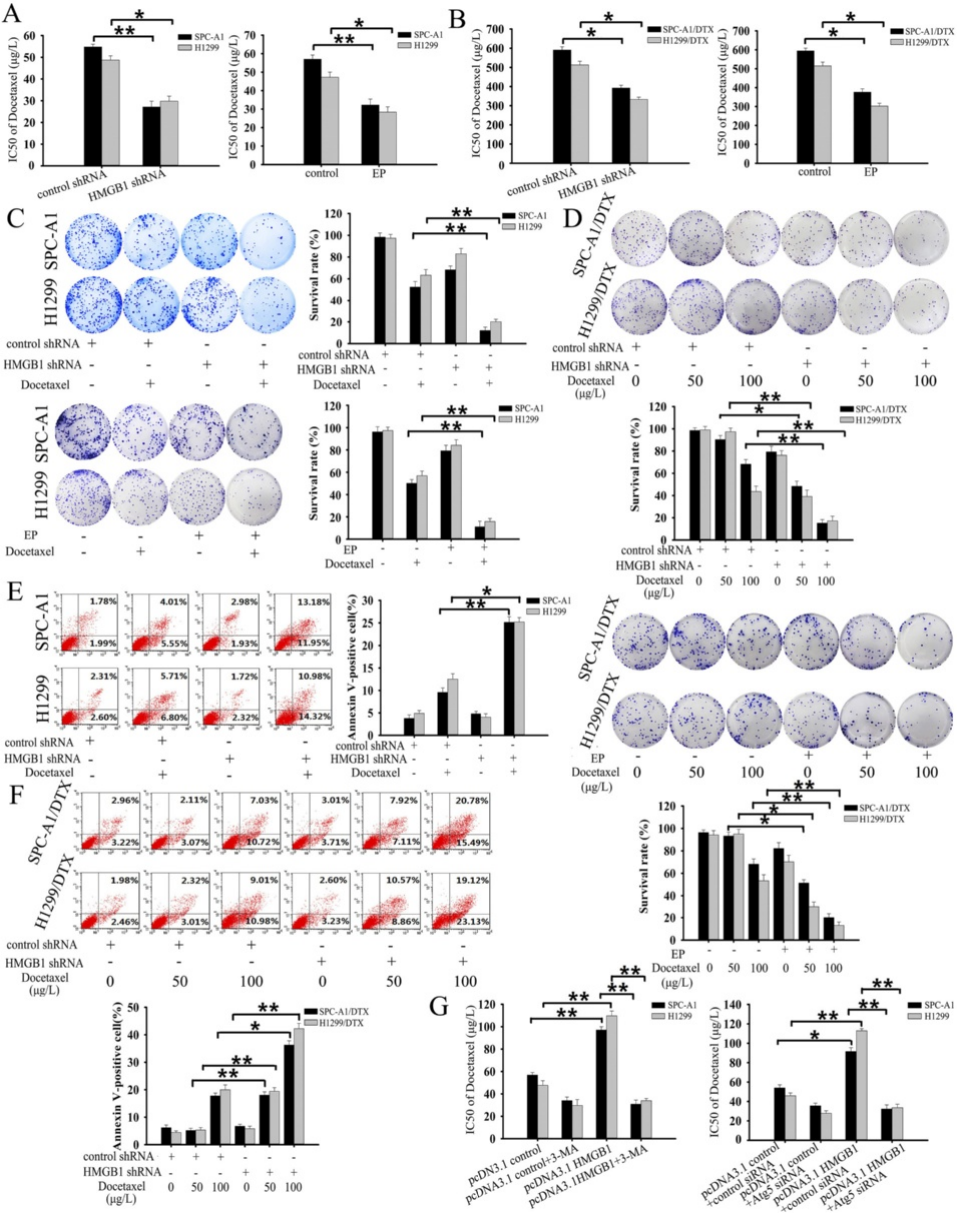
**Docetaxel induced autophagy in LAD cells. (A)** SPC-A1 and H1299 cells were treated with either the indicated concentrations of docetaxel for 24 h or 10 μg/l docetaxel for varying periods. Whole cell lysates were subjected to western blot analysis for LC3, p62 and GAPDH expression (as a loading control). **(B)** Both parental cells (SPC-A1 and H1299) and docetaxel-resistant cells (SPC-A1/DTX and H1299/DTX) were transiently transfected with a GFP-LC3 construct. Twenty-four hours later, parental cells were exposed to docetaxel (10 μg/l) for an additional 24 h. GFP-LC3 dot formation was analyzed as described in Materials and Methods (mean ± S.D. of three independent experiments, *P *<* 0.05, **P *<* 0.01). Bar = 50 μm. **(C)** SPC-A1 and H1299 cells pretreated with or without bafilomycin A1 (20 nM, 2 h) were exposed to docetaxel (10 μg/l) for an additional 24 h. Whole cell lysates were analyzed by western blot. **(D, E)** Parental cells (SPC-A1 and H1299) and docetaxel-resistant cells (SPC-A1/DTX and H1299/DTX) were analyzed by **(D)** western blot analysis for LC3 and GAPDH expression and **(E)** transmission electron microscopy analysis, as described in Materials and methods. A magnified view of the electron photomicrograph showed the characteristic autophagosomes. N: nucleus. The results were obtained from three independent experiments.

To assess the effect of docetaxel on autophagy and the role of autophagy in determining the sensitivity of LAD cells to docetaxel, first we evaluated autophagy activity in SPC-A1 and H1299 cells after treatment with docetaxel. As shown in Figure 1A, western blot analysis revealed that docetaxel treatment led to a dose- and time-dependent increase in the level of LC3-II and decrease in the amount of p62, two selective markers of autophagy, in both SPC-A1 and H1299 cells. The effect of docetaxel on autophagy was next confirmed by a GFP-LC3 punctate formation assay [15]. After transfecting both cell lines with a GFP-LC3 plasmid, we observed an abundance of punctate fluorescent dots after 24 h docetaxel treatment, which reflects the conversion from cytoplasmic LC3-I to the phosphatidylethanolamine-conjugated form LC3-II (Figure 1B). To further demonstrate the activation of autophagy by docetaxel, we treated cells with an autophagy-lysosomal inhibitor, bafilomycin A1, 2 h prior to docetaxel treatment [15,16]. As displayed in Figure 1C, in the presence of bafilomycin A1, LC3-II levels were increased in both cell lines in comparison with docetaxel alone treatment. We then analyzed autophagy activity in two docetaxel-resistant cell lines, SPC-A1/DTX and H1299/DTX, which were previously established in our lab. The baseline levels of LC3-II were higher in the docetaxel-resistant lines than in the parental cells (Figure 1D). SPC-A1/DTX and H1299/DTX showed increased autophagy activity as measured by analysis of GFP-LC3 punctate formation using confocal microscopy (Figure 1B) and by ultrastructural analysis of characteristic autophagosomes using transmission electron microscopy (Figure 1E).

**Figure 2:**
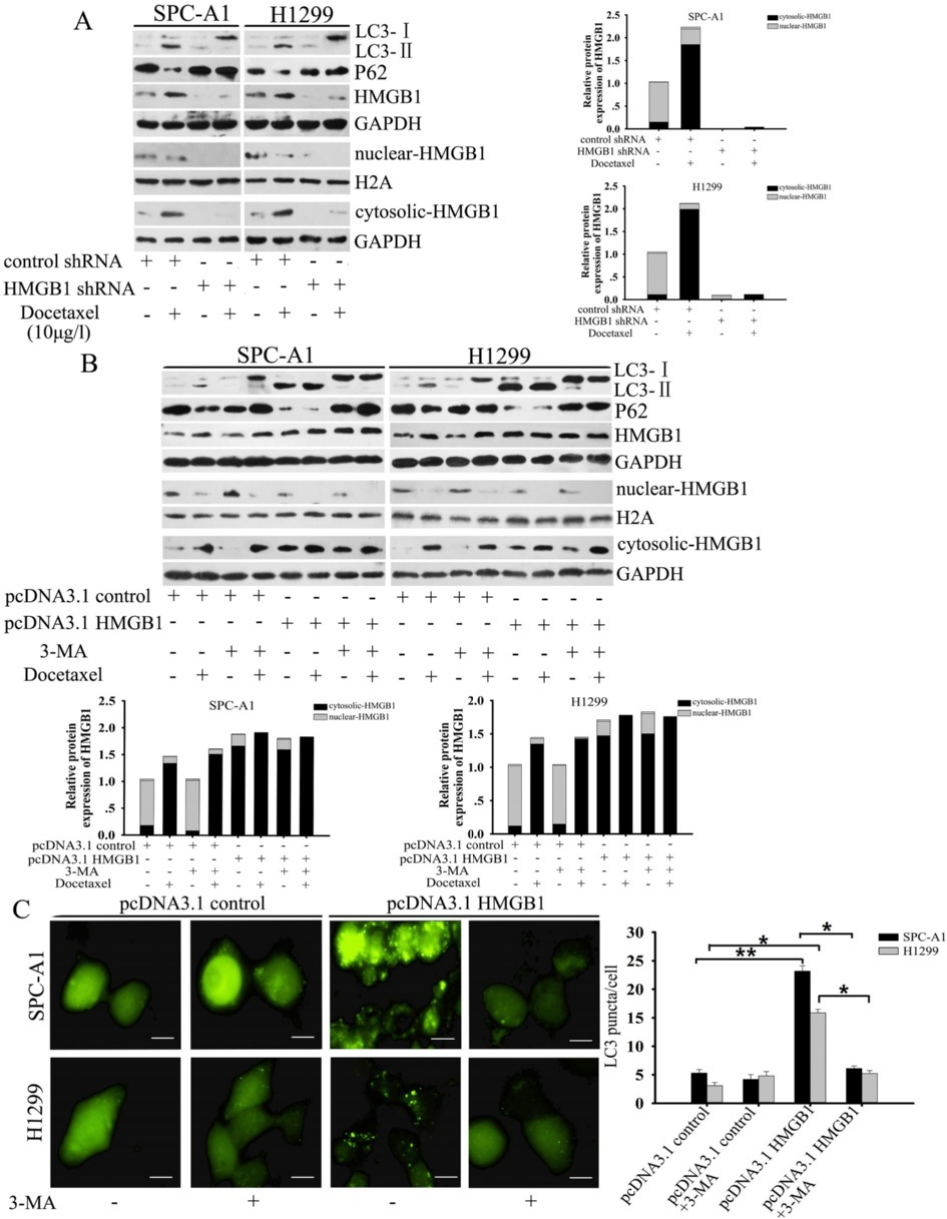
**Inhibition of autophagy enhanced sensitivity of LAD cells to docetaxel. (A, B)** LAD cells were treated with 3-methyladenine (3-MA, 5 mM) or transiently transfected with Atg5 or control siRNA. Cells were exposed to the indicated concentrations of docetaxel for 48 h. Viable cells were determined with an MTT assay as described in Materials and methods. **(C, D)** LAD cells were incubated with various concentrations of docetaxel for 48 h in the presence or absence of 3-MA. The colonies were stained and counted, and survival curves were constructed from three independent experiments. **(E)** SPC-A1 and H1299 cells were treated with docetaxel (10 μg/l) in the presence or absence of 3-MA (5 mM, 2 h) or Atg5 siRNA. Apoptosis was determined by flow cytometric analysis of Annexin-V/PI staining. **(F)** SPC-A1/DTX cells were treated with the indicated doses of docetaxel in the presence or absence of 3-MA or Atg5 siRNA. Apoptosis was determined by flow cytometric analysis of Annexin-V/PI staining. Each point or bar represents mean ± S.D. of triplicate determinations. *P*<* 0.05, **P< 0.01 compared with docetaxel alone treatment.

Next, to determine whether docetaxel-induced autophagy played a protective or resistant role in the drug treatment, we treated SPC-A1 and H1299 cells with an autophagy inhibitor, 3-methyladenine (3-MA), or transfected cells with small-interfering RNA (siRNA) specifically targeting the autophagic gene Atg5 before addition of docetaxel. Both 3-MA and Atg5 siRNA efficiently blocked activation of autophagy, which caused an increase of cytotoxicity and apoptosis and decrease in the proliferation rate of SPC-A1 and H1299 cells (Figure 2A, 2C and 2E). Western blot analysis of c-caspase3 and c-PARP also confirmed the results (Additional file 1: Figure S1A and S1B). However, the sensitivity of SPC-A1/DTX and H1299/DTX cells to docetaxel was markedly enhanced (Figure 2B) while the proliferation rate was greatly diminished (Figure 2D) after addition of 3-MA or silencing of Atg5. Moreover, both docetaxel-resistant cell lines showed an increased propensity for apoptosis after inhibition of autophagy (Figure 2F, Additional file 1: Figure S1C and S1D).

Taken together, these finding suggests that autophagy is induced by docetaxel and functions as a docetaxel resistance mechanism in LAD cells. Therefore, suppression of autophagy might be a novel strategy for circumventing the resistance of LAD cells to docetaxel.

### Docetaxel promoted HMGB1 expression and cytosolic translocation

**Figure 3:**
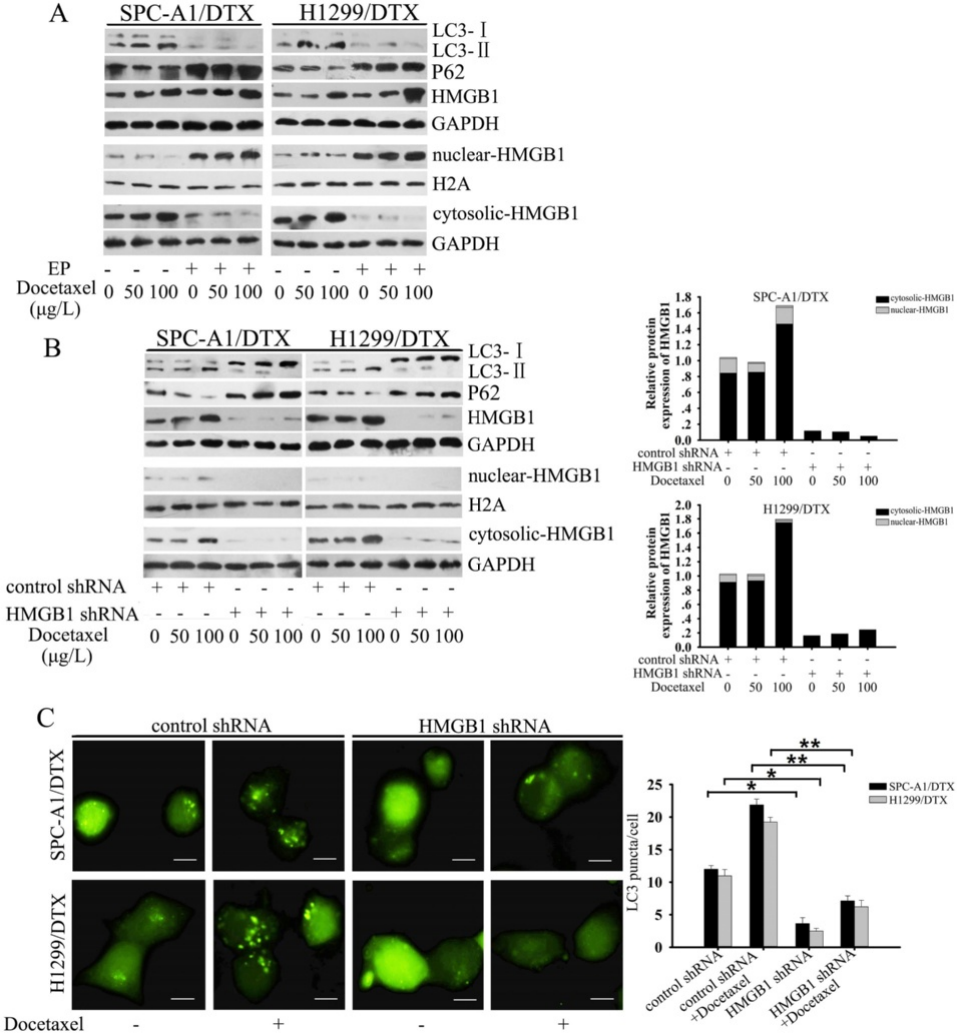
**Docetaxel promoted HMGB1 expression and cytosolic translocation. (A)** SPC-A1 cells were treated with docetaxel (10 μg/l) for the indicated periods. Total cell lysates, nuclear extracts, cytoplasmic fractions and extracellular medium were prepared and HMGB1 levels were analyzed by western blot. **(B)** SPC-A1 cells were pretreated with or without ethyl pyruvate (EP, 10 mM, 1 h) before addition of docetaxel (10 μg/l) for 48 h. Whole cell lysates, nuclear extracts and cytoplasmic fractions were analyzed by western blot for HMGB1. **(C)** SPC-A1 cells transfected with pcDNA3.1-HMGB1 or control vector were treated with EP (10 mM, 1 h). Total cell lysates, nuclear extracts, cytoplasmic fractions were analyzed by western blot for HMGB1. **(D)** Total cell lysates, nuclear extracts, and cytoplasmic fractions from parental and docetaxel-resistance LAD cells were analyzed by western blot for HMGB1. **(E)** Western blot analysis of HMGB1 in the presence or absence of EP (10 mM, 1 h) in SPC-A1/DTX and H1299/DTX cells. GAPDH was used as a loading control for whole cell lysates, extracellular medium and cytoplasmic extracts, and H2A was used as a loading control for nuclear extracts. The experiments were performed in triplicate. H1299 cells were subjected to the analyses as above and the results are shown in Additional file 2: Figure S2.

To explore the potential role of HMGB1 in the regulation of docetaxel-induced autophagy, we analyzed HMGB1 protein expression and location in LAD cells exposed to docetaxel (10 μg/l) for indicated periods of time. Western blot analysis of nuclear and cytosolic fractions of SPC-A1 and H1299 cells indicated that in the absence of treatment, HMGB1 was mainly located in the nucleus, with very low levels in the cytoplasm. Docetaxel markedly enhanced total levels of HMGB1 in both parental cells in a time-dependent manner. Moreover, following exposure to docetaxel, cytosolic levels were elevated at 6 h and kept rising until 48 h, while the nuclear levels were reduced at the same time. Accumulation of LC3-II showed an obvious elevation after treatment with docetaxel for 6 h, and continued to increase until 48 h (Figure 3A and Additional file 2: Figure S2A). Notably, ethyl pyruvate (EP), a pharmacological inhibitor of HMGB1 cytoplasmic translocation [7], attenuated docetaxel-induced autophagy, as shown in Figure 3B and Additional file 2: Figure S2B. Furthermore, diminished cytosolic HMGB1 also limited the activation of autophagy even in cells transfected with cDNA encoding full-length human HMGB1 (Figure 3C and Additional file 2: Figure S2C).The level of cytosolic HMGB1 exhibited a positive correlation with docetaxel-induced autophagy. Meanwhile, SPC-A1/DTX and H1299/DTX cells also showed relatively higher total level of HMGB1 which included a higher cytosolic level and a lower nuclear level under basal conditions compared to parental cells (Figure 3D). However, application of EP decreased LC3-II accumulation (Figure 3E).

### HMGB1 altered sensitivity of LAD cells to docetaxel

**Figure 4:**
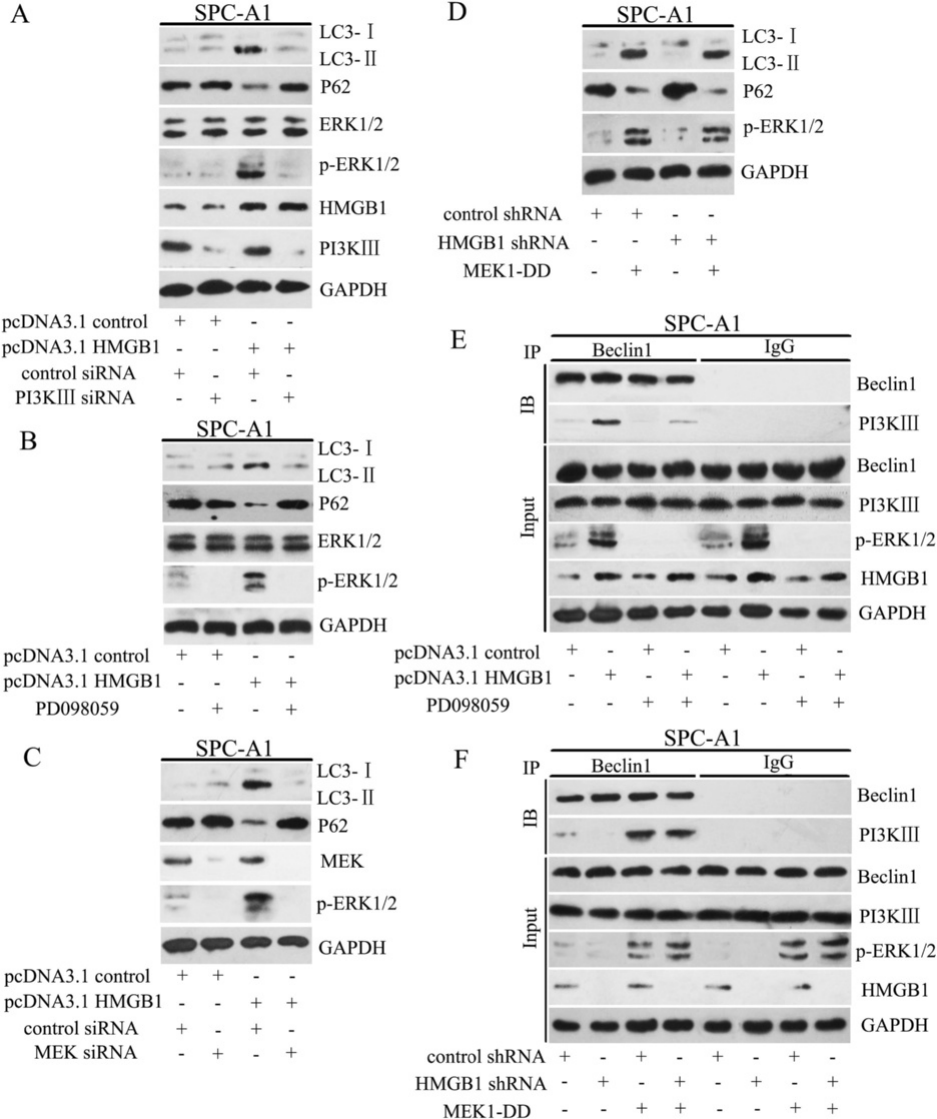
**HMGB1 altered the sensitivity of LAD cells to docetaxel *in vitro*.** Parental and docetaxel-resistant LAD cells were transfected with HMGB1 shRNA or pretreated with EP before incubation with indicated concentrations of docetaxel for 48 h. **(A, B)** Cell viability was analyzed by MTT assay. **(C, D)** Cell proliferation was evaluated by colony formation assay. **(E, F)** Apoptosis was analyzed by flow cytometric analysis of Annexin-V/PI staining. **(G)** SPC-A1 cells transfected with pcDNA3.1 control or pcDNA3.1-HMGB1 were exposed to indicated doses of docetaxel for 48 h in the presence or absence of 3-MA (5 mM) or Atg5 siRNA. Cell viability was analyzed by MTT assay. The results shown are the representative of three identical experiments, and the bars are the mean ± S.D. *P < 0.05, **P < 0.01.

To clarify the role of HMGB1 in LAD cells following chemotherapy, we analyzed the responses of SPC-A1 and H1299 cells to docetaxel treatment with depleted for HMGB1 expression. Knockdown of HMGB1 or limiting HMGB1 cytosolic translocation in SPC-A1 and H1299 cells enhanced the cellular response to docetaxel (Figure 4A) and inhibited cell proliferation (Figure 4C). Docetaxel induced apoptotic cell death to a great extent after HMGB1 knockdown, as shown by an increase of Annexin-V positive cells by 15.59 ± 1.01% and 12.79 ± 0.88% in SPC-A1 and H1299 cells, respectively (Figure 4E), as well as increased level of activated caspase3 and PARP cleavage (Additional file 3: Figure S3A). Moreover, treatment with an apoptosis inhibitor, Z-VAD-fmk [17], reduced the activation of caspase3 (Additional file 3: Figure S3A). Conversely, pretreatment with HMGB1 increased drug resistance in both parental LAD cells, as displayed by an elevated cell survival rate. Nevertheless, blockade of autophagy by 3-MA or Atg5 siRNA reversed HMGB1-induced protection against docetaxel (Figure 4G).

The effect of HMGB1 gene silencing on enhancing docetaxel sensitivity was even more biologically significant in the docetaxel-resistant cells. Similar to the results after pharmacologically inhibiting autophagy in Figure 2A, transfection of SPC-A1/DTX and H1299/DTX cells with HMGB1 shRNA rendered them largely more sensitive to docetaxel, as indicated by a decrease in the number of surviving cells (Figure 4B) and increase of apoptosis (Additional file 3: Figure S3B). The proliferation rate was also significantly reduced (Figure 4D). Importantly, addition of EP caused similar effects as knockdown of HMGB1 (Figure 4B).

### HMGB1 modulated docetaxel-induced autophagy in LAD cells

**Figure 5:**
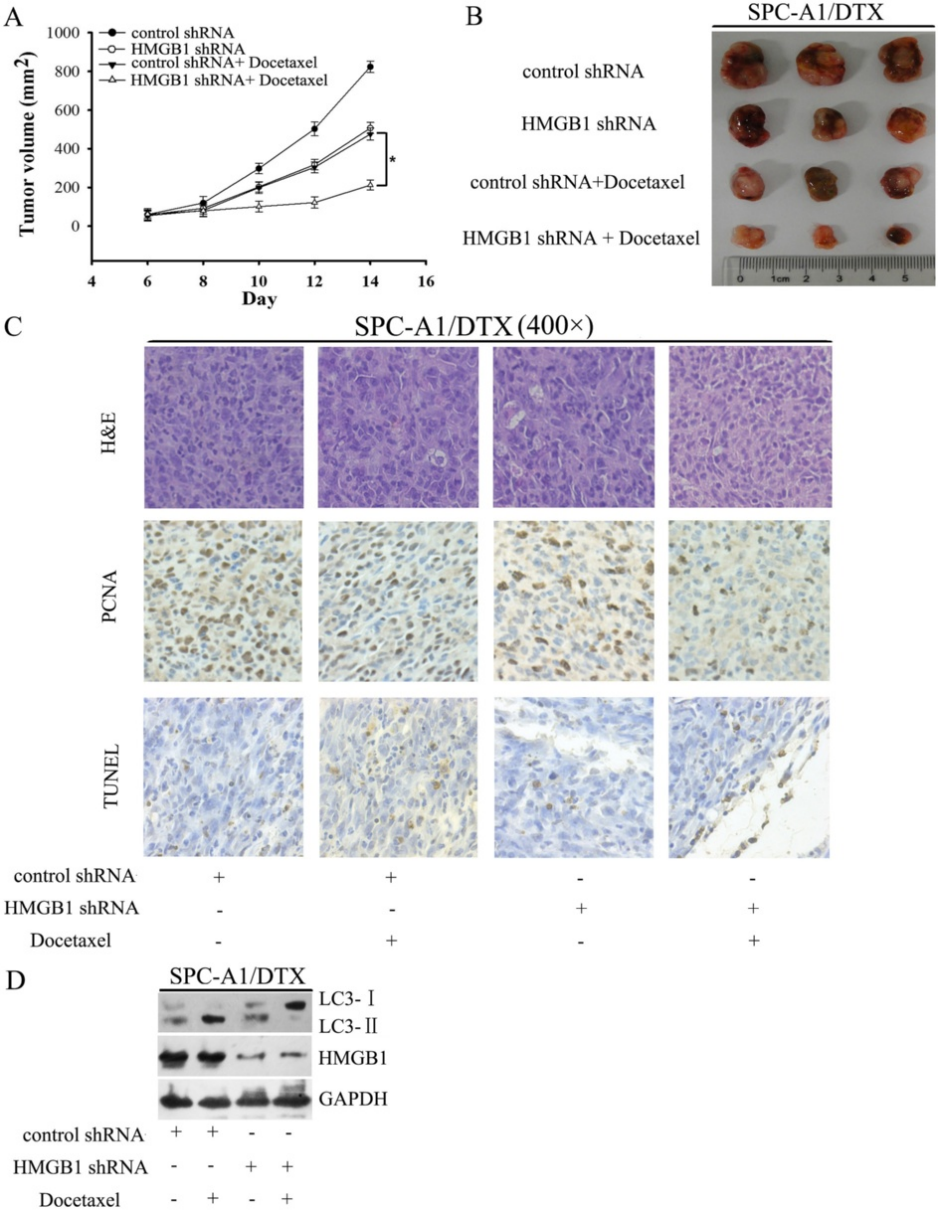
**HMGB1 regulated docetaxel-induced autophagy in parental LAD cells. (A)** Whole cell lysates, nuclear exacts and cytoplasmic fractions from both parentalcells transfected with control or HMGB1 shRNA followed by various concentrations of docetaxel treatment for a further 48 h were subjected to western blot analysis for LC3, p62 and HMGB1. **(B)** SPC-A1 cells transfected with control or pcDNA3. 1-HMGB1 were treated with or without 3-MA (5 mM, 2 h) before addition of docetaxel (10 μg/l). Total cell lysates, nuclear extracts, and cytoplasmic fractions were subjected to western blot analysis for LC3, p62 and HMGB1. **(C)** SPC-A1 cells were co-transfected with either control or pcDNA3.1-HMGB1 and GFP-LC3 plasmid in the presence or absence of 3-MA (5 mM, 2 h). Bar = 50 μm. Values are reported as mean ± S.D. of three independent experiments. *P < 0.05, **P < 0.01.

**Figure 6:**
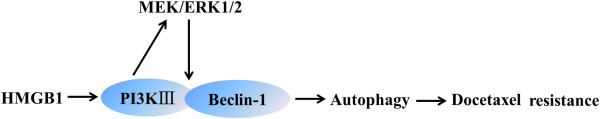
**HMGB1 regulated docetaxel-induced autophagy in docetaxel-resistant LAD cells. (A)** Whole cell lysates, nuclear exacts and cytoplasmic fractions from SPC-A1/DTX and H1299/DTX cells were treated with indicated doses of docetaxel in the presence or absence of EP(10 mM, 1 h) were subjected to western blot analysis for LC3, p62 and HMGB1. **(B) **Both docetaxel-resistant cells were transfected with HMGB1 shRNA before treatment with the indicated doses of docetaxel for 48 h. Total cell lysates, nuclear extracts, cytoplasmic fractions were subjected to western blot analysis for LC3, p62 and HMGB1. **(C)** Both docetaxel-resistant cells were co-transfected with either control or HMGB1 shRNA and GFP-LC3 plasmid followed by treatment with docetaxel (100 μg/l) for an additional 24 h. Bar = 50 μm. Values are reported as mean ± S.D. of three independent experiments. *P < 0.05, **P < 0.01.

To investigate whether HMGB1 is a direct activator of autophagy, we evaluated the LC3-I to LC3-II conversion and LC3 punctate formation by fluorescent analysis as described above. Knockdown of HMGB1 by transfecting SPC-A1 and H1299 cells with HMGB1 shRNA prevented the appearance of LC3-II and the degradation of p62 after docetaxel treatment (Figure 5A). Similarly, downregulation of HMGB1 expression or inhibition of cytosolic translocation of HMGB1 with EP decreased the level of LC3-II in SPC-A1/DTX and H1299/DTX cells, and a high dose of docetaxel failed to induce LC3-II turnover to a greater degree (Figure 6A and 6B). In contrast, overexpression of HMGB1 significantly increased LC3-II accumulation and autophagic p62 degradation. However, this elevated conversion of LC3-I to LC3-II could be abolished by suppression of autophagy with 3-MA (Figure 5B). Fluorescence micrographs showed a punctate pattern of GFP-LC3 in HMGB1-overexpressing SPC-A1 and H1299 cells, which could be greatly attenuated by application of 3-MA (Figure 5C). Meanwhile, compared with the vector control groups, SPC-A1/DTX and H1299/DTX cells transfected with HMGB1 shRNA exhibited reduced formation of GFP-LC3 punctate staining after docetaxel treatment (Figure 6C). These data support a vital role for HMGB1 in the regulation of autophagy in LAD cells.

### mTORC1 dependent pathway was not required for HMGB1-mediated autophagy in LAD cells

Accumulating evidence has demonstrated that inhibition of the Akt/mTORC1 pathway is linked to the trigger for autophagy [18,19]. mTOR, a downstream effector of Akt, plays a critical role in the regulation of protein synthesis by phosphorylation of p70S6K (70 kDa ribosomal protein S6 kinase) [20]. Thus, we sought to test whether HMGB1 could regulate docetaxel-induced autophagy through the Akt/mTORC1 pathway in LAD cells.

SPC-A1 cells overexpressing HMGB1 showed no distinct change in the level of phosphor-Akt(Ser473), phosphor-mTOR(Ser2448), phosphor-S6RP (Additional file 4: Figure S4A), nor did the docetaxel-resistant SPC-A1/DTX cells transfected with HMGB1 shRNA (Additional file 4: Figure S4B).

Suppression of mTORC1 activity is considered as a crucial step in the initiation of autophagy [21]. Additionally, rapamycin, a specific inhibitor of mTORC1 [22], enhanced the accumulation of LC3-II in SPC-A1 cells transfected with the control vector but not in cells transfected with HMGB1 shRNA (Additional file 4: Figure S4C). In addition, siRNA-mediated silencing of mTORC1 conferred less protection in HMGB1 knockdown cells due to lessened autophagy activity (Additional file 4: Figure S4D). Collectively, these results indicated that HMGB1 might not regulate autophagy through a mTORC1-dependent pathway in LAD cells.

### HMGB1 promoted Beclin-1-PI3K-III core complex formation through the MEK/ERK1/2 pathway

**Figure 7:**
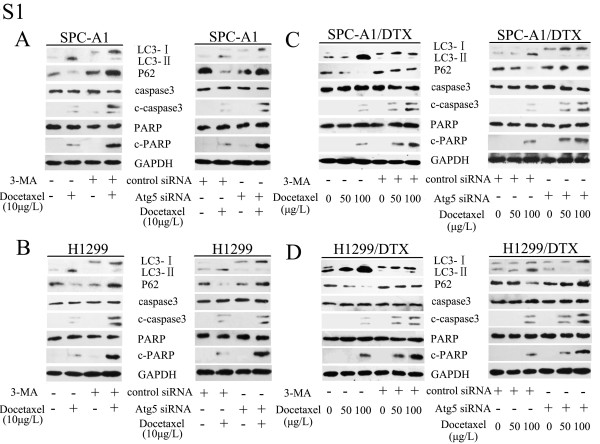
**HMGB1 promoted Beclin-1-PI3K-III core complex formation through the MEK/ERK1/2 pathway. (A)** SPC-A1 cells were transfected with pcDNA3.1-HMGB1, PI3K-III siRNA or both for 48 h. Total cell lysates were subjected to western blot analysis of LC3, p62, p-ERK1/2 and GAPDH (as a loading control). **(B, E)** SPC-A1 cells were pretreated with PD098059 (20 μg/ml, 45 min), followed by transfection with control or pcDNA3.1-HMGB1. Whole cell lysates were analyzed by **(B)** western blot against LC3, p62, and p-ERK1/2 and **(E)** immunoprecipitation (IP) as described in Materials and methods. **(C)** SPC-A1 cells were transfected with pcDNA3.1-HMGB1, MEK siRNA or both for 48 h. Total cell lysates were examined by western blot analysis using specific antibodies against LC3, p62, MEK and p-ERK1/2. **(D, F)** SPC-A1 cells were transfected with control or HMGB1 shRNA, and then infected with the constitutively active MEK1 construct, MEK1-DD. Total cell lysates were analyzed by **(D)** western blot for LC3, p62 and p-ERK1/2 and **(F)** IP. The blots shown are representative of three separate experiments in which similar results were observed.

As shown in Figure 5B, we found that 3-MA, a potent PI3K inhibitor, markedly inhibited HMGB1-induced LC3-II conversion. Thus, we tested whether PI3K-III, which had been reported to help promote autophagy initiation [4,23], was required for HMGB1-induced autophagy. To exclude the effect of 3-MA on other kinases and cellular processes, siRNA specifically targeting PI3K-III was transfected into SPC-A1 cells. Figure 7A showed that reduction of PI3K-III expression in these cells distinctly inhibited HMGB1-induced autophagy, implying that PI3K-III is required for HMGB1-mediated autophagy.We next evaluated the role of the MEK-ERK1/2 pathway in HMGB1-induced autophagy, in which MEK functions as an immediate upstream activator of ERK1/2. Transfection of SPC-A1 cells with an HMGB1 cDNA plasmid activated the MEK-ERK1/2 pathway by increasing the phosphorylation of ERK1/2. However, pharmacological disruption of the MEK-ERK1/2 pathway by the specific inhibitor PD098059 that prevents ERK1/2 activation (Figure 7B) or by transfection with MEK siRNA (Figure 7C) reduced HMGB1-induced LC3-II formation and p62 degradation. Notably, suppression of PI3K-III also inhibited HMGB1-induced phosphorylation of the MEK-ERK1/2 pathway, suggesting that MEK-ERK1/2 is a downstream signal from PI3K-III (Figure 7A). Moreover, transfection of SPC-A1 cells with MEK1-DD, the constitutively active form of MEK1, an upstream activator of ERK, relieved autophagy inhibition mediated by HMGB1 (Figure 7D).

The Beclin-1-PI3K-III core complex is essential for vesicle nucleation in autophagic stages [24]. Transfection with pcDNA3.1-HMGB1 enhanced the formation of the Beclin-1-PI3K-III complex. However, addition of PD098059 blocked the interaction between Beclin1 and PI3K-III in SPC-A1 cells (Figure 7E). Conversely, transfection of the active MEK construct, which conferred resistance to the suppression of MEK-ERK1/2 pathway phosphorylation, eliminated the negative control of Beclin-1-PI3K-III complex formation by HMGB1 (Figure 7F). These studies suggest that the MEK-ERK1/2 pathway is required for HMGB1-induced formation of the Beclin-1-PI3K-III complex.

### Downregulation of HMGB1 enhanced the response of SPC-A1/DTX cells to docetaxel *in vivo*

**Figure 8:**
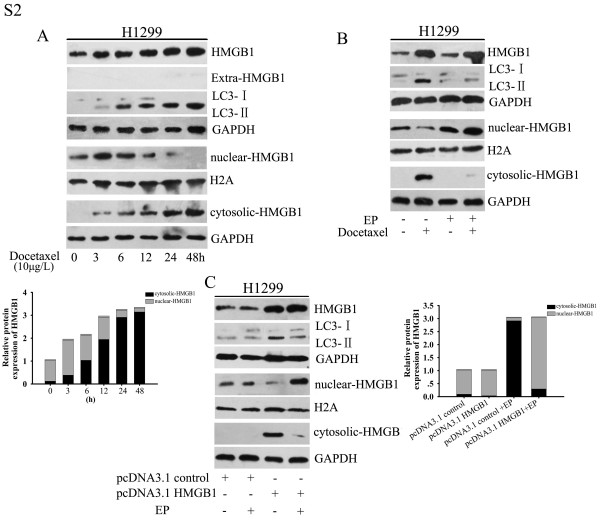
**Suppression of HMGB1 resensitized docetaxel-resistant SPC-A1/DTX cells to docetaxel *in vivo. *(A)** NOD/SCID mice were inoculated with 5.0 × 10^6^ SPC-A1/DTX cells following transfection of control or HMGB1 shRNA and treated with docetaxel (1 mg/kg) beginning at day 6. Tumor volumes were calculated for 14 days. (n = 5; *P < 0.05). **(B)** Representative photographs of tumors formed at 2 weeks after subcutaneous transplantation are shown. **(C)** Hematoxylin and eosin (H&E)-stained, proliferating cell nuclear antigen (PCNA)-stained and TUNEL assay stained sections of the transplanted tumors are shown (original magnification, ×400). **(D)** HMGB1 and LC3 expression in the transplanted tumors were determined by western blot analysis.

To test the effects of targeted downregulation of HMGB1 on chemosensitivity of LAD cells *in vivo*, we inoculated NOD/SCID mice with SPC-A1/DTX cells transfected with HMGB1 shRNA. Tumors derived from HMGB1 shRNA-transfected SPC-A1/DTX cells grew more slowly compared with those derived from control shRNA-transfected cells after being treated with docetaxel (Figure 8A and B). Immunohistochemistry analysis showed that the positive rate of PCNA in the HMGB1 shRNA-transfected group was greatly diminished (Figure 8C). TUNEL staining of resected tumor tissues also revealed even lower apoptosis in the HMGB1 shRNA group than the control group. We also observed a decreased level of LC3-II expression in HMGB1 shRNA-transfected tumors in response to docetaxel in comparison with control shRNA-transfected tumors (Figure 8D). These results support the critical role of HMGB1 in modulating the chemosensitivity in docetaxel-resistant LAD cells *in vivo*.

## Discussion

In this study, we demonstrated that HMGB1-regulated autophagy is a significant contributor to docetaxel resistance in LAD cells. Suppression of HMGB1 or inhibition HMGB1 cytosolic translocation diminished autophagic protection in response to docetaxel. In addition, we found that HMGB1 promoted Beclin-1-PI3K-III core complex formation through the MEK/ERK1/2 pathway.

A variety of mechanisms underlying drug resistance have been well established, such as DNA repair mechanisms, drug export transporters and resistance to apoptosis [25]. Cancer cells exhibit multiple responses to chemotherapy, including the initiation of cell survival pathways and the activation of cell death pathways. Although previous studies considered autophagic cell death as an alternative form of cell death due to excessive self-digestion in the absence of apoptosis, overwhelming evidence supports the idea that autophagy functions primarily as a cell survival mechanism, especially when cells are subjected to various stresses associated with cell death [6]. Consistent with the documented cytoprotective role of autophagy in a number of cancer cells, here we proved that blockade of autophagy potentiated docetaxel-induced cell death in SPC-A1 and H1299 cells and resensitized the docetaxel-resistant SPC-A1/DTX and H1299/DTX cells with a higher baseline level of LC3-II to docetaxel to some extent. Our results clearly revealed that autophagic activation is highly related to docetaxel resistance in LAD cells. A detailed understanding of autophagy regulation in LAD might contribute to predict and overcome chemoresistance, thereby improving therapeutic efficacy.

HMGB1 is generally located inside the nucleus in normally proliferating cells, and is released to the cytoplasm and even extracellular space following different stimuli, such as starvation, metabolic stress and chemotherapy, in innate immune cells [26] as well as several types of cancer cell lines [13,14]. Both endogenous and exogenous HMGB1 have been considered as vital regulators of autophagy in tumor cells [7,27]. Our focus here was the interaction between endogenous HMGB1 and autophagy. We observed that docetaxel promoted HMGB1 expression. Interestingly, we found a consistency in the timing between cytosolic HMGB1 translocation and the elevation of autophagic activity during the entire time course of docetaxel treatment, suggesting that subcellular localization of HMGB1 may correlate with autophagy induction. Cytosolic translocation of HMGB1 as an origin of autophagy has been demonstrated in other human cell types *in vitro* and *in vivo* under various cytotoxic stresses [28,29]. Our results showed that the basal level of autophagy in docetaxel-resistant cells was decreased after suppressing HMGB1 cytosolic translocation or knockdown of HMGB1. However, disrupting HMGB1 cytosolic translocation had no apparent effect on autophagy induction, even in HMGB1-overexpressing LAD parental cells. These results proved that cytosolic translocation of HMGB1 is likely a cause rather than an effect of autophagy in LAD cells treated with docetaxel. In support of this notion, inhibition of autophagy failed to abolish the increase of cytosolic HMGB1 levels. HMGB1 functions as a pro-autophagic protein, while autophagy also regulates release of HMGB1 following cytotoxic stress [8]. Nevertheless, we detected no obvious increase in the level of HMGB1 in the extracellular environment. The reason for failure of autophagy to enhance HMGB1 release in LAD cells exposed to docetaxel remains unclear, but it is conceivable that the regulation between autophagy and HMGB1 is cell type-dependent and may also be related to the agent in question.

Overexpression of HMGB1 is associated with six hallmarks of cancer, including self-sufficiency in growth signals and insensitivity to inhibitors of growth [30]. Depletion of HMGB1 greatly enhanced the sensitivity to antitumor agents [31,32]. Consistent with these findings, we confirmed that HMGB1 serves as a positive regulator of autophagy and mediates docetaxel resistance. Furthermore, inhibition of the cytosolic translocation of HMGB1 had similar effects on cytotoxicity and autophagy disruption as knockdown of HMGB1 in docetaxel-resistant cells, suggesting that cytosolic HMGB1 causes autophagic activation, which results in resistance to docetaxel. As a pro-survival protein, HMGB1 promotes cancer growth and development [33]. In our study, reduced HMGB1 significantly inhibited tumor growth following docetaxel treatment *in vivo*. Together these data suggest that HMGB1 serves as a positive regulator of autophagy and mediates the docetaxel resistance.

Emerging evidence has proven the role of Akt/mTORC1 pathway inhibition in the promotion of autophagy. However, we found that HMGB1 expression had only negligible effects on the activity of Akt/mTORC1 signaling both in SPC-A1 and SPC-A1/DTX cells. Moreover, suppression of mTORC1 promoted autophagy in the control vector group but not in the HMGB1-overexpressed group. Autophagic stages, including induction, vesicle nucleation, vesicle elongation and completion, are controlled by a series of autophagy genes regulated by specific signaling molecules [23]. Numerous investigations have indicated that mTORC1 serves as a negative regulator of autophagy induction [21]. However, our data found that inhibition of mTORC1 failed to eliminate the effects of HMGB1 suppression on LC3-II conversion, suggesting that the mTORC1-dependent pathway may not be required for HMGB1-mediated autophagy and that HMGB1 might regulate downstream autophagic steps.

Furthermore, HMGB1 promotes Beclin-1-PI3K-III complex formation, potentially through the MEK/ERK1/2 pathway. The PI3K family consists of three classes: I, II, and III. PI3K-III activity is required for autophagic activation, while PI3K-I has a negative effect on autophagy [5]. Beclin1 recruits PI3K-III to form the Beclin-1-PI3K-III complex, thus triggering autophagosome nucleation [24]. However, our results added another role for PI3K-III in HMGB1-mediated autophagy. We demonstrated that HMGB1 regulated autophagy through activating the MEK/ERK1/2 pathway, while the genetic inhibition of PI3K-III deprived the HMGB1-induced phosphorylation of the MEK-ERK1/2 pathway and inhibited autophagic activation. These results suggest that MEK/ERK1/2 signaling functions as a downstream signal of PI3K-III in HMGB1-induced autophagy. The correlation between PI3K and ERK was previously reported to regulate the early onset of inflammatory pain [34]. An early study demonstrated that co-targeted PI3K and MEK signaling pathways enhanced cytotoxicity of histone deacetylase inhibitors in NSCLC and chronic myeloid leukemia [35].

**Figure 9:**
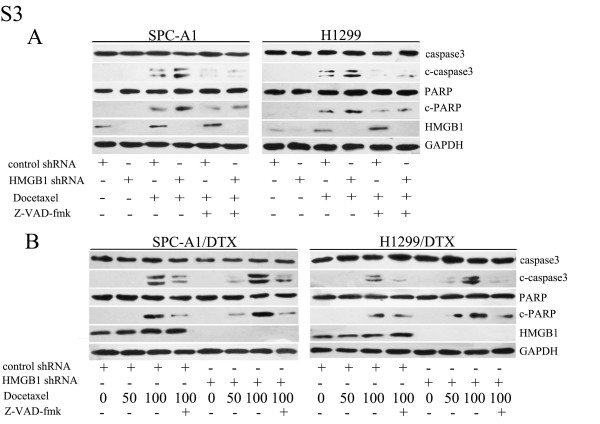
**Model depicting the mechanism by which HMGB1 modulates docetaxel resistance by regulating autophagy.** Docetaxel promotes the cytosolic translocation of HMGB1. Cytosolic HMGB1 acts as an activator of autophagy, which potentiates the formation of the Beclin-1-PI3K-III complex through activating the MEK/ERK1/2 signaling pathway. PI3K-III also serves as an upstream signal of MEK/ERK1/2 for facilitating the core complex formation.

In addition, we verified that the activation of the MEK-ERK1/2 signaling pathway was involved in HMGB1-mediated formation of the Beclin-1-PI3K-III complex. HMGB1 was shown to bind with Beclin-1, which then promotes Beclin-1-PI3K-III complex formation [28]. However, the assembly of the complex appears to differ in cell- and/or tissue-dependent manners [36]. A previous study indicated that ULK1-FIP200 complex formation is required for the interaction of this complex in osteosarcoma cells [37]. We found that inhibition of HMGB1 or MEK also limited the interaction between Beclin-1 and PI3K-III. However, the negative regulation of the Beclin-1- PI3K-III complex mediated by HMGB1 can be reversed after upregulation of MEK activity. Together with data from the mechanistic studies, we propose a model in which HMGB induces Beclin-1-PI3K-III complex formation through activating the MEK/ERK1/2 signaling pathway. Moreover, PI3K-III also serves as an upstream signal of MEK/ERK1/2 for facilitating the core complex formation in HMGB1-regulated autophagy (Figure 9).

In summary, here we showed translocation of HMGB1 from the nucleus to the cytoplasm in response to docetaxel in LAD cells, which acts as a positive regulator of autophagy that leads to diminished apoptosis and increased drug resistance. HMGB1 promotes the formation of Beclin-1-PI3K-III complex through activating the MEK/ERK1/2 signaling pathway, thereby regulating autophagosome formation. Our results support future investigation of HMGB1 as a strategic target for LAD therapy.

## Materials and methods

### Cell lines and reagents

Human LAD cell lines SPC-A1 and H1299 were purchased from the Tumor Cell Bank of Chinese Academy of Medical Science (Shanghai, China) and cultured in RPMI 1640 medium containing 10% fetal bovine serum and ampicillin and streptomycin at 37°C in a humidified atmosphere of 95% air and 5% CO_2_. Docetaxel-resistant SPC-A1 and H1299 cell lines (SPC-A1/DTX and H1299/DTX) were established and preserved in 50 μg/L final concentration of docetaxel in our laboratory. Antibodies against GAPDH, LC3, p62, caspase3, activated (cleaved) caspase3 (c-caspase3), PARP, cleaved PARP (c-PARP), Atg5, HMGB1, mTOR, phosphorylated mTOR (p-mTOR), Akt, p-Akt, S6RP, p-S6RP, MEK, ERK1/2, p-ERK1/2, and H2A were obtained from Cell Signaling Technology. Bafilomycin A1, 3-methyladenine (3-MA), ethyl pyruvate (EP) and PD098059 were purchased from Sigma Aldrich (St. Louis, MO).

### cDNA constructs, siRNA and transfection

The GFP-tagged LC3 cDNA expression construct was a gift from Dr. Noboru Mizushima (Tokyo Medical and Dental University, Tokyo, Japan). P-Babe-Puro-MEK-DD, which constitutively expresses activated MEK1-DD (S218D/S222D), was purchased from Addgene Inc. (Cambridge, MA). Transfections with pcDNA3.1-HMGB1, HMGB1 shRNA, Atg5 siRNA, mTOR siRNA, PI3K-III siRNA and MEK siRNA (all obtained from GenePharma, Shanghai, China) were performed using Lipofectamine 2000 (Invitrogen, USA), according to the manufacturer’s protocol.

### Cell viability

Cells were cultured in 96-well plates with 100 μl medium per well and treated with the indicated drug combination for 48 h. MTT (5 mg/ml, 20 μl) was added to each well and cells were incubated for 4 h at 37°C. The media was removed and 100 μl of DMSO was added per well to solubilize the formazan product. The relative number of surviving cells in each group was determined by measuring the optical density (O.D.) of the cell lysates at 560 nm.

### Colony formation assay

Cells were plated in triplicate (800 cells/well) in 6-well plates for approximately 24 h under standard conditions. After specific treatments, the cells then were exposed to various doses of docetaxel. After 14 days of incubation, the colonies were fixed with methanol, stained with 0.5% crystal violet in absolute ethanol, and colonies with ≥50 cells were counted under a dissection microscope. In each irradiation dose group, the surviving fraction of cells was calculated as plating efficiency of the irradiated cells divided by the plating efficiency of the irradiated cells by that of mock control.

### Preparation of subcellular fractions and western blot analysis

Cytosolic extracts, nuclear extracts, extracellular medium and total cellular lysates were prepared using the NE-PER nuclear and cytoplasmic extraction kit (Piece, Rockford, USA) according to the manufacturer’s instructions. Protein concentrations of the extracts were measured with BCA assay (Pierce, Rockford, USA) and equalized with the extraction reagents. Equal amount of the extracts were loaded and subjected to SDS-PAGE, transferred onto nitrocellulose members, and analyzed as described previously [38].

### Immunoprecipitation analysis

Cells were lysed at 4°C in RIPA buffer (Millipore). Samples containing equal amounts of proteins were precleared with protein A sepharose (Millipore) and subsequently incubated with irrelevant immunoglobulin or specific antibodies in the presence of protein A sepharose beads. The beads were washed three times with RIPA buffer, and the immune complexes were eluted from the beads and subjected to SDS-PAGE and western blot analysis.

### Apoptosis assay

We measured apoptosis using an Annexin-V-fluorescein isothiocyanate apoptosis detection kit (Oncogene Research Products, Boston, MA) that quantitatively measures the percentage of early apoptotic cells via flow cytometric analysis. In addition, western blot analysis for c-PARP and c-caspase3 after various treatments was performed. The degree of apoptosis in tissue was assessed with the TUNEL kit (Roche) according to the manufacturer’s instructions.

### GFP-LC3 analysis

Cells were transfected with a GFP-LC3-expressing plasmid. After 24 h, cells were fixed in 3.7% formaldehyde for 20 min, washed with PBS, mounted and inspected using a fluorescence microscope. A minimum of 150 GFP-positive cells were counted under each condition, and the graphs were plotted as percentage of GFP-LC3 positive cells over total transfected cell population.

### Transmission electron microscopy

Cells were fixed with a solution containing 3% glutaraldehyde plus 2% paraformaldehyde in 0.1 mol/L phosphate buffer (pH 7.4), followed by 1% OsO_4._ After dehydration, thin sections were stained with uranyl acetate and lead citrate for observation under a JEM 1011CX electron microscope (JEOL, USA, Inc.) Digital images were obtained using an Advanced Microscopy Techniques imaging system.

### Mice xenograft models and immunohistochemistry analysis

All animal experiments strictly followed the guidelines of the Institutional Review Board of Jinling Hospital. Approximately 5.0 × 10^6^ SPC-A1/DTX/shcontrol or SPC-A1/DTX/ shHMGB1 cells were suspended in 100 μl PBS and injected subcutaneously into the right side of the posterior flank of female BALB/c athymic nude mice (Department of Comparative Medicine, Jinling Hospital, Nanjing, China) at 5 to 6 weeks of age. Tumor volumes were examined every other day and were calculated using the equation: V = A × B^2^/2(mm^3^), where A is the largest diameter and B is the perpendicular diameter. When the average tumor size reached approximately 50 mm^3^, docetaxel was administered via intraperitoneal injection at a dose of 1 mg/kg at one dose every other day with for three total doses [39]. After 2 weeks, all mice were killed, and necropsies were performed. The primary tumors were excised and analyzed by hematoxylin and eosin (H&E) staining, immunohistochemistry staining of proliferating cell nuclear antigen (PCNA) [40], TUNEL staining [41] and western blot analysis for LC3 and HMGB1 protein expression.

### Statistical analyses

Statistical analyses and data plotting were performed using SigmaPlot software version 12. Results were presented as mean ± S.D. of three independent experiments and analyzed with the Student’s *t* test. *P* < 0.05 was considered statistically significant for all analyses.

## Abbreviations

LAD: Lung adenocarcinoma; NSCLC: Non-small cell lung cancer; HMGB1: High-mobility group box 1; MEK: Mitogen-activated protein kinase; ERK: Extracellular signal-regulated kinase; 3-MA: 3-methyladenine; siRNA: Small interfering RNA; EP: Ethyl pyruvate; p70S6K: 70 kDa ribosomal protein S6 kinase; H&E: Hematoxylin and eosin; PCNA: Proliferating cell nuclear antigen.

## Competing interests

The authors declare that they have no competing interests.

## Authors’ contributions

LC and HS designed and guided the study. BP and DC performed all the cytology tests and molecular biology experiments. JH performed western blot analysis and immunoprecipitation analysis. RW established the mice xenograft models and performed immunohistochemistry analysis. BF collected and analyzed the data. BP drafted the manuscript. All authors read and approved the final manuscript.

## Supplementary Material

Additional file 1: Figure S1 Inhibition of autophagy enhanced apoptosis of LAD cells in response to docetaxel. (A, B) SPC-A1 and H1299 cells were treated with docetaxel (10 μg/l) in the presence or absence of 3-methyladenine (3-MA, 5 mM, 2 h) or Atg5 siRNA. Western blot analyzed the expression of LC3, p62, cleaved-PARP (c-PARP) and cleaved caspase3 (c-caspase3). (C, D) SPC-A1/DTX and H1299/DTX cells were treated with indicated doses of docetaxel in the presence or absence of 3-MA or Atg5 siRNA. Whole cell lysates were subjected to western blot analysis of LC3, p62, c-PARP and c-caspase3 . GAPDH was used as an internal control. Click here for file

Additional file 2: Figure S2 Docetaxel promoted HMGB1 expression and cytosolic translocation. (A) H1299 cells were treated with docetaxel (10 μg/l) for the indicated periods. Total cell lysates, nuclear extracts, cytoplasmic fractions and extracellular medium were prepared and HMGB1 levels were analyzed by western blot. (B) H1299 cells were pretreated with or without ethyl pyruvate (EP, 10 mM, 1 h) before addition of docetaxel (10 μg/l) for 48 h. Whole cell lysates, nuclear extracts and cytoplasmic fractions were analyzed by western blot for HMGB1. (C) H1299 cells transfected with pcDNA3.1-HMGB1 or control vector were treated with EP (10 mM, 1 h). Total cell lysates, nuclear extracts, cytoplasmic fractions were analyzed by western blot for HMGB1. GAPDH was used as a loading control for whole cell lysates, extracellular medium and cytoplasmic extracts, and H2A was used as a loading control for nuclear extracts. The experiments were performed in triplicate. Click here for file

Additional file 3: Figure S3 Knockdown of HMGB1 increased apoptosis of LAD cells in response to docetaxel. After transfection with control or HMGB1 shRNA for 48 h, (A) parental and (B) docetaxel-resistant LAD cells were exposed to docetaxel (50 μg/l and 100 μg/l) for an additional 48 h with or without Z-VAD-fmk (20 μmol/L, 1 h) pretreatment. Apoptosis was evaluated by western blot analysis of c-PARP and c-caspase3. Click here for file

Additional file 4: Figure S4 mTORC1-dependent pathway was not required for HMGB1-mediated autophagy. (A) SPC-A1 cells with overexpressed HMGB1 and (B) SPC-A1/DTX cells silenced for HMGB1 were subjected to western blot analysis of p-Akt(Ser473), p-mTOR(Ser2448) and p-S6RP. (C) SPC-A1 cells were pretreated with or without rapamycin (50 mM, 2 h) before transfection with control or HMGB1 shRNA. (D) SPC-A1 cells were co-transfected with either control or HMGB1 shRNA and mTORC1 siRNA. Whole cell lysates were subjected to western blot analysis of p-mTOR(Ser2448), LC3 and p62. GAPDH was used as a sample loading control. The figures show a representative experiment of three separate experiments with similar results. Click here for file
