# Supplementary figures and images for: HMGB1-mediated autophagy promotes docetaxel resistance in human lung adenocarcinoma
Source: Mol Cancer. 2014 Jul 5;13:165. doi: 10.1186/1476-4598-13-165 (PMC4125709; doi:10.1186/1476-4598-13-165)

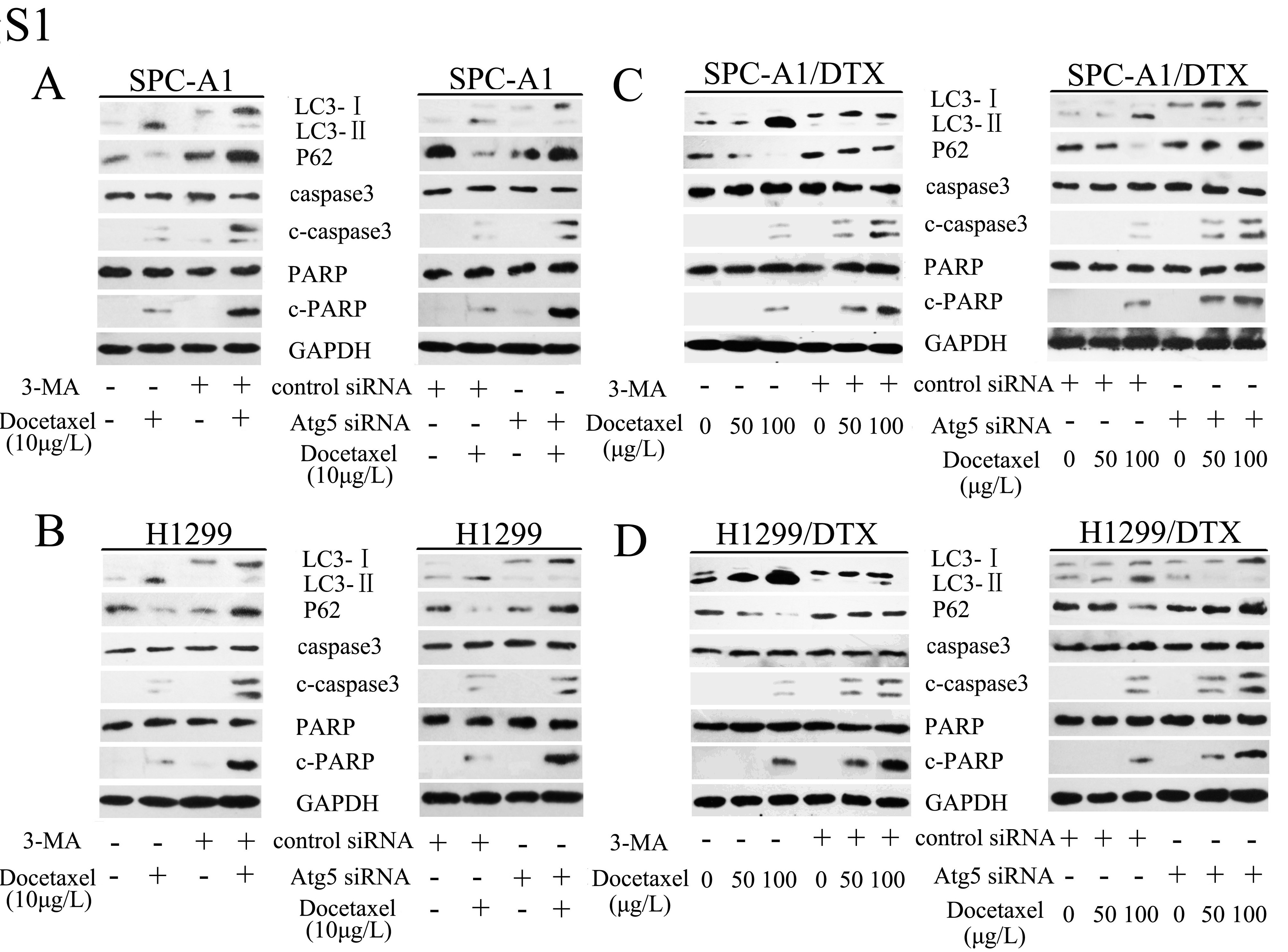

Supplement: Additional file 1: Figure S1 — Inhibition of autophagy enhanced apoptosis of LAD cells in response to docetaxel. (A, B) SPC-A1 and H1299 cells were treated with docetaxel (10 μg/l) in the presence or absence of 3-methyladenine (3-MA, 5 mM, 2 h) or Atg5 siRNA. Western blot analyzed the expression of LC3, p62, cleaved-PARP (c-PARP) and cleaved caspase3 (c-caspase3). (C, D) SPC-A1/DTX and H1299/DTX cells were treated with indicated doses of docetaxel in the presence or absence of 3-MA or Atg5 siRNA. Whole cell lysates were subjected to western blot analysis of LC3, p62, c-PARP and c-caspase3 . GAPDH was used as an internal control. [file 1476-4598-13-165-S1.tiff]

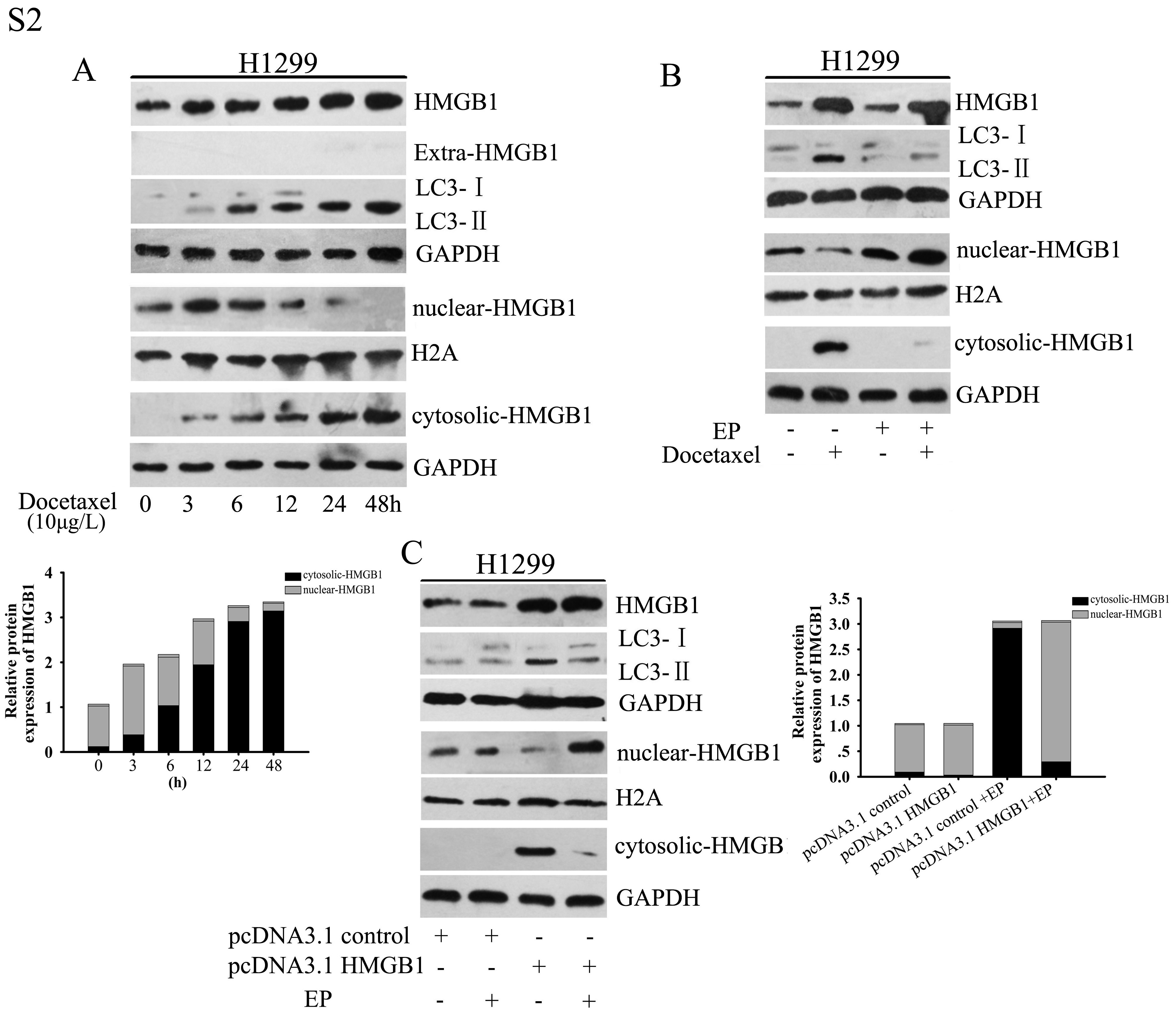

Supplement: Additional file 2: Figure S2 — Docetaxel promoted HMGB1 expression and cytosolic translocation. (A) H1299 cells were treated with docetaxel (10 μg/l) for the indicated periods. Total cell lysates, nuclear extracts, cytoplasmic fractions and extracellular medium were prepared and HMGB1 levels were analyzed by western blot. (B) H1299 cells were pretreated with or without ethyl pyruvate (EP, 10 mM, 1 h) before addition of docetaxel (10 μg/l) for 48 h. Whole cell lysates, nuclear extracts and cytoplasmic fractions were analyzed by western blot for HMGB1. (C) H1299 cells transfected with pcDNA3.1-HMGB1 or control vector were treated with EP (10 mM, 1 h). Total cell lysates, nuclear extracts, cytoplasmic fractions were analyzed by western blot for HMGB1. GAPDH was used as a loading control for whole cell lysates, extracellular medium and cytoplasmic extracts, and H2A was used as a loading control for nuclear extracts. The experiments were performed in triplicate. [file 1476-4598-13-165-S2.tiff]

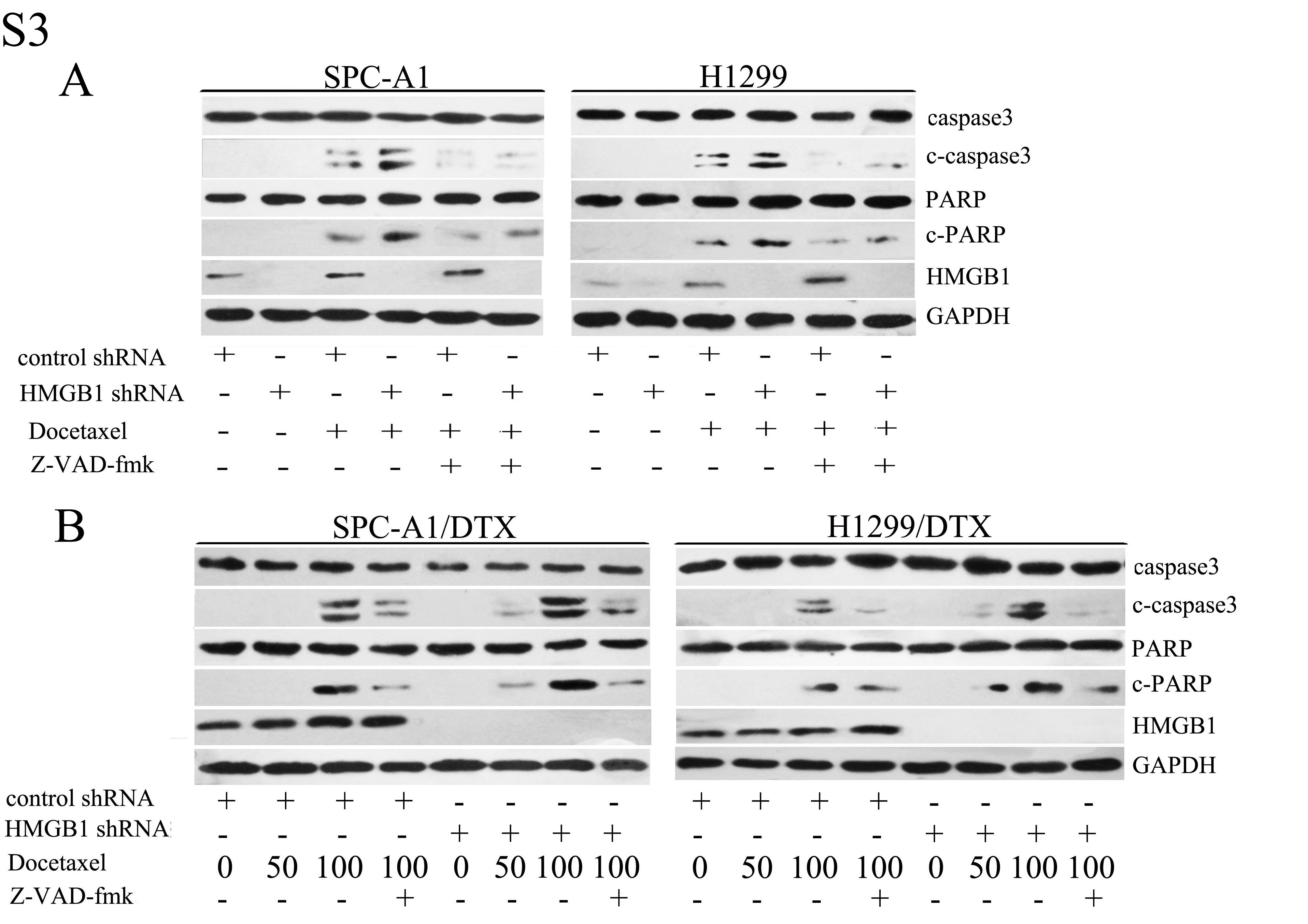

Supplement: Additional file 3: Figure S3 — Knockdown of HMGB1 increased apoptosis of LAD cells in response to docetaxel. After transfection with control or HMGB1 shRNA for 48 h, (A) parental and (B) docetaxel-resistant LAD cells were exposed to docetaxel (50 μg/l and 100 μg/l) for an additional 48 h with or without Z-VAD-fmk (20 μmol/L, 1 h) pretreatment. Apoptosis was evaluated by western blot analysis of c-PARP and c-caspase3. [file 1476-4598-13-165-S3.tiff]

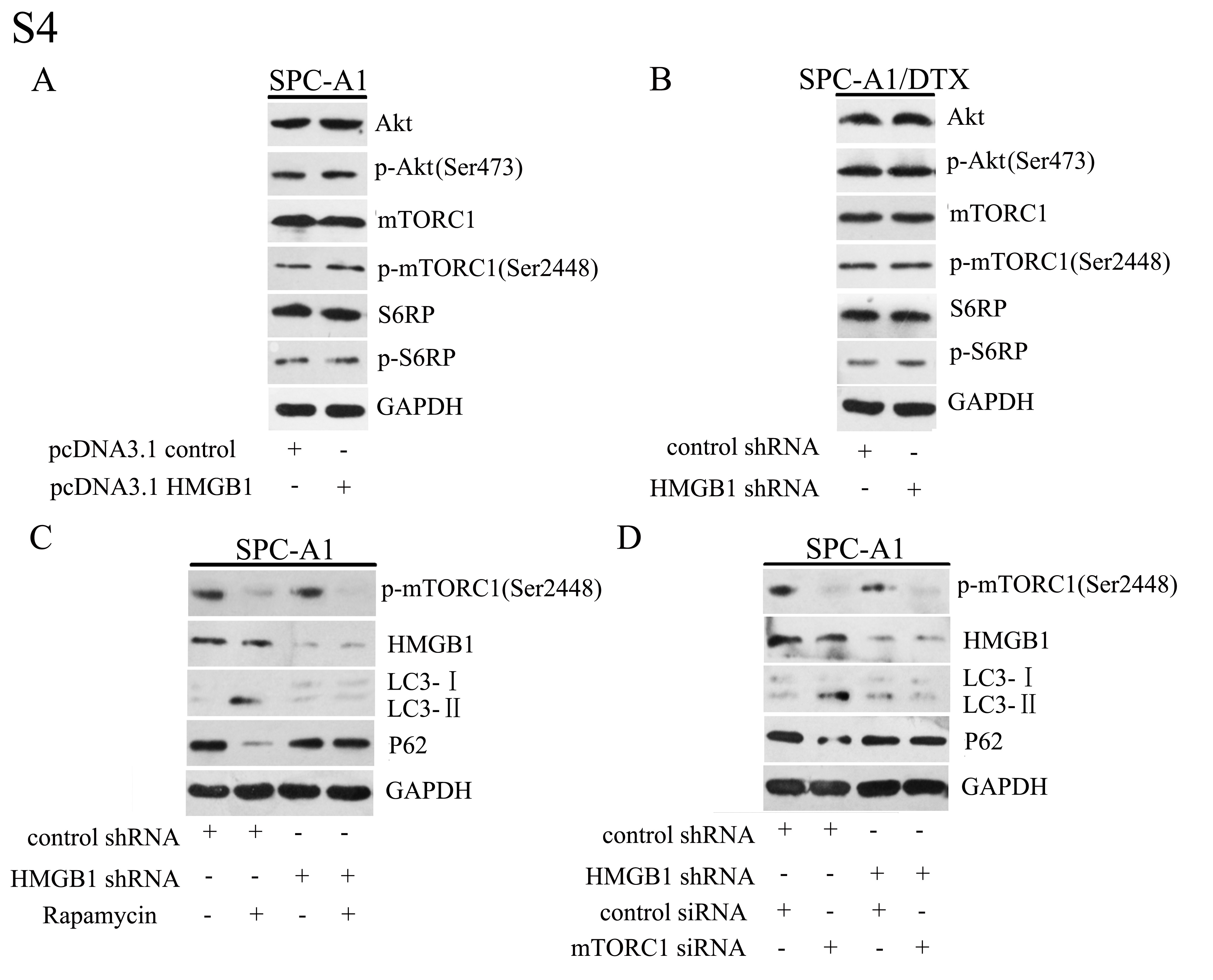

Supplement: Additional file 4: Figure S4 — mTORC1-dependent pathway was not required for HMGB1-mediated autophagy. (A) SPC-A1 cells with overexpressed HMGB1 and (B) SPC-A1/DTX cells silenced for HMGB1 were subjected to western blot analysis of p-Akt(Ser473), p-mTOR(Ser2448) and p-S6RP. (C) SPC-A1 cells were pretreated with or without rapamycin (50 mM, 2 h) before transfection with control or HMGB1 shRNA. (D) SPC-A1 cells were co-transfected with either control or HMGB1 shRNA and mTORC1 siRNA. Whole cell lysates were subjected to western blot analysis of p-mTOR(Ser2448), LC3 and p62. GAPDH was used as a sample loading control. The figures show a representative experiment of three separate experiments with similar results. [file 1476-4598-13-165-S4.tiff]
